# Supplementary material for: Color image encryption algorithm based on ∞-shaped transformation and closed-loop control model
Source: PLoS One. 2026 Mar 31;21(3):e0345460. doi: 10.1371/journal.pone.0345460 (PMC13038027; doi:10.1371/journal.pone.0345460)
Supplement: Attachment — Submitted filename: Response to Reviewers.docx [file pone.0345460.s002.docx]

**Response to Reviewers “Color Image Encryption Algorithm Based on ∞-Shaped Transformation and Closed-Loop Control model ”**

Feng Zhao, Xiaoqiang Zhang, and Fang Zhu

Revised version for PLOS One, February 2026

**Feb 14, 2026**

**Dear Prof. Haris Calgan,**

We would like to express our sincere thanks for your giving us the valuable opportunity to revise our manuscript. Meanwhile, thanks to three anonymous reviewers for their constructive comments.

The revised version of our manuscript has been modified based on their helpful comments. All the revisions to the manuscript are marked in red font. In the following correspondence, we describe the changes made in this version. We hope that now the paper is ready for publication.

Looking forward to hearing your positive response soon.

Happy New Year! Best wishes,

Your sincerely,

Prof. Feng Zhao, Prof. Xiaoqiang Zhang, Prof. Fang Zhu

**Response to Reviewer 1’s Comments**

Firstly, we would like to express our sincere thanks for your constructive comments and positive response. The revised version of the manuscript has been significantly modified based on your comments. The revised parts are marked in red font. We hope you are satisfied with our revisions.

1. In Eq. (3), the term pi-squared divided by x(n-1) is problematic. If the state variable x approaches zero during iteration, the system will overflow or become unstable. You must discuss how to handle this singularity or prove that the trajectory never hits zero in the defined phase space.

**ANS:** As you noted, according to Eq. (3), *x*_n_∈[−1,1] and *y*_n_∈[−1,1]. As a result, singularities may occur during the iterative process when *x*_n−1_=0. In practical numerical iterations, a regularization method is adopted to address this issue: when |*x*_n-1_|< *ε*, its value is adjusted to $x_{n-1}\leftarrow x_{n-1}+\mathrm{sgn}( x_{n-1}) \cdot\varepsilon^{'}$, where *ε*′ is set to 10^−10^. This approach effectively avoids divergence caused by singularity. Since only a negligible perturbation is introduced, the overall chaotic dynamics of the

system remain largely unchanged.

2. The proposed 2D-CSHS involves complex transcendental functions (cosine, sine, exponential). Compared to simple Logistic or Tent maps, this computational overhead is high. You should provide a specific floating-point operation count (FLOPs) analysis or comparison to justify using such a heavy map for image encryption.

**ANS:** To build a more secure and complex image encryption system, this paper designs a novel two-dimensional chaotic system based on the composition of trigonometric and nonlinear functions. Although this system has higher computational complexity compared to classical Logistic or Tent maps, such design is essential to ensure the encryption strength. To quantify its computational demand, we conducted a floating-point operation (FLOPs) analysis on this map. Each iteration requires approximately 12 fundamental floating-point operations (including 4 exponentiations, 4 multiplications, 2 trigonometric functions, and 2 additions). Although the computational overhead is higher than that of one-dimensional maps, a single iteration can still be completed within nanoseconds on modern general-purpose processors, posing no substantial impact on the overall encryption efficiency. More importantly, this system overcomes the inherent limitations of one-dimensional chaotic maps in encryption, such as their small key space and insufficient security. By expanding the parameter space and enhancing the unpredictability of orbits, it significantly improves the security of image encryption while still maintaining real-time performance.

3. The definition in Eq. (6) Beta = P * Alpha suggests matrix multiplication. If you implement this as actual matrix multiplication, the complexity is O(N^2), which is unacceptable for large images. You must clarify if this is implemented via coordinate mapping O(N) and strictly define the index generation rules.

**ANS:** The definition in Equation (6) is a matrix multiplication. However, there are five types of such transformations in the algorithm, the largest of which involves a matrix of 3 rows and 5 columns. Correspondingly, the transformation matrix P is only of size 15×15, meaning the computational complexity of this matrix operation is on the order of *N*(15^2^ ). This complexity does not vary with image size; it primarily depends on the maximum transformation matrix used.

4. Why is the ∞-shaped path superior to established paths like Hilbert, ZigZag, or Peano curves? The paper only shows the path visually. You need to provide a metric (e.g., degree of scrambling or distance between adjacent pixels) to compare your method against classical scanning curves.

**ANS:** The ∞-shaped transform differs from conventional methods such as the Hilbert transform, Zigzag transform, or Peano curve transform in two main aspects. First, it utilizes an inherent three-row matrix transformation to directly achieve inter-channel scrambling among the three color channels. Second, it is divided into five distinct forms based on different column counts, enabling dynamic scrambling effects that effectively enhance the complexity of the scrambling algorithm.

To verify the effectiveness of the ∞-shaped transform compared to classical scrambling methods such as Zigzag, simulation experiments were conducted using a 256×256 house image, with the results presented in Table 3. It presents a comparative analysis of correlation coefficients for both adjacent pixels and inter-channel pixels under different scrambling schemes. It can be observed that the ∞-shaped transform outperforms the Hilbert transform, Zigzag transform, and Peano curve transform in disrupting both spatial correlations among neighboring pixels and statistical dependencies across color channels. Specifically, this scheme substantially reduces the horizontal, vertical, and diagonal correlations of the R, G, and B channels, while also effectively weakening the inter-channel correlations (e.g., R–G, R–B, G–B). These results demonstrate the superior decorrelation capability and scrambling randomness of the ∞-shaped transform. In contrast, the other scrambling methods exhibit limited effectiveness in suppressing inter-channel correlation, with some cases showing negligible impact. Overall, the ∞-shaped transform achieves more comprehensive

performance in image scrambling tasks.

**Table 3.  Comparison of correlation coefficients of different scrambling schemes.**

| **Scrambling Path** | | | **Original**  **image** | **Hilbert transform** | **Zigzag transform** | **Peano curve transform** | **∞-shaped transform** |
| --- | --- | --- | --- | --- | --- | --- | --- |
| **Adjacent Pixel Correlation** | R | H | 0.9671 | 0.6984 | 0.1980 | 0.7679 | 0.6638 |
|  |  | V | 0.9353 | 0.6975 | 0.1977 | 0.6504 | 0.6696 |
|  |  | D | 0.9126 | 0.5613 | 0.1661 | 0.5504 | 0.5659 |
|  | G | H | 0.9805 | 0.7614 | 0.1669 | 0.7899 | 0.7685 |
|  |  | V | 0.9474 | 0.7598 | 0.1674 | 0.7247 | 0.7643 |
|  |  | D | 0.9320 | 0.6367 | 0.1478 | 0.6240 | 0.5254 |
|  | B | H | 0.9820 | 0.8190 | 0.2039 | 0.8404 | 0.7258 |
|  |  | V | 0.9749 | 0.8180 | 0.2055 | 0.7881 | 0.7318 |
|  |  | D | 0.9625 | 0.7101 | 0.2061 | 0.6903 | 0.7404 |
| **Inter-channel Pixel Correlation** | R-G | | 0.6378 | 0.6378 | 0.6378 | 0.6378 | 0.5420 |
|  | R-B | | 0.4823 | 0.4823 | 0.4823 | 0.4823 | 0.6300 |
|  | G-B | | 0.9418 | 0.9418 | 0.9418 | 0.9418 | 0.6499 |

5. In Section 5.8 (Robustness), the decrypted images are clear despite noise/cropping. However, your diffusion is “closed-loop”, which typically implies a strong avalanche effect (one bit change affects all). If the algorithm is robust to noise, the diffusion might be weak. Please explain this contradiction theoretically.

**ANS:** As you have rightly noted, there exists a certain trade-off between robustness and diffusion capability. Generally, if the diffusion avalanche effect is strong, robustness tends to be weaker, whereas a weaker avalanche effect often corresponds to better robustness. However, based on our research group’s analysis and discussions, we believe that robustness is not solely determined by diffusion but is also closely related to scrambling. In this paper, we propose an image encryption scheme based on an infinite-type transformation diffusion method and a closed-loop control model. Experimental results confirm its excellent encryption performance. In future algorithm research, we will continue to examine the balance between robustness and diffusion avalanche effects, striving to achieve an optimal equilibrium that meets practical application requirements.

6. The key space calculation in Section 5.1 assumes a precision of 10^-15 translates directly to effective key bits. This is a common mistake. In IEEE 754 double-precision standard, the effective entropy is limited (approx 52 bits mantissa). Please revise the calculation based on actual computer representation standards.

**ANS:** We sincerely thank the reviewer for identifying this critical inaccuracy in our original key space calculation. The reviewer is absolutely correct. Our previous method of equating a decimal precision of 10^−15^ directly to 10^15^ possible values was incorrect, as it ignores the finite representation of floating-point numbers in digital computers.

Inspired by your suggestion, we have completely revised the key space analysis in Section 5.1. The new calculation is now based on the IEEE 754 double-precision floating-point standard. We conservatively assume that each floating-point initial value or parameter provides approximately 52 bits of effective entropy (corresponding to the 52-bit mantissa).The key space for the form dependent on floating-point values is now calculated as (2^52^)^8^=2^416^.The overall effective key space of the algorithm is taken as this value (2^416^), which remains far above the 2^100^ threshold required to resist brute-force attacks.

We have updated the text, removed the incorrect calculation based on 10^−15^ precision, and cited the relevant standard. We believe the revised analysis is now technically sound and accurately reflects the algorithm’s security against exhaustive key search.

7. In Tables 5 and 6 (NPCR/UACI), you only list “passed”. This is not scientific. You must list the actual P-values or the specific critical values corresponding to the image size and significance level (alpha=0.05) used in your hypothesis testing to prove the pass. Please refer to Two-dimensional coupling-enhanced cubic hyperchaotic map with exponential parameters: construction, analysis, and application in hierarchical significance-aware multi-image encryption for revisions.

**ANS:** Inspired by your suggestions, after carefully reading the recommended literature, we have thoroughly revised and rewritten the calculation standards and experimental results of the NPCR and UACI metrics in Section 5.7 “Differential Attack Analysis” of the paper. The revised content has been highlighted in red within the text.

8. While Figure 13 shows flat histograms, visual inspection is insufficient for a high-quality journal. You should calculate and tabulate the Chi-square statistics for the cipher images to quantitatively demonstrate the uniformity of the pixel distribution. Please refer to State-dependent variable fractional-order hyperchaotic dynamics in a coupled quadratic map: a novel system for high-performance image protection for revisions.

**ANS:** Inspired by your suggestions,We have supplemented the content of Section 5.4 “Histogram Analysis” by adding experiments on the chi-square test of pixel values in the encrypted images. The calculation results show that all encrypted images passed the test, which provides more objective evidence of the encryption algorithm’s capability to resist statistical analysis attacks.

9. Table 9 shows encryption takes ~7.5 seconds for a 1024x1024 image. This is quite slow for practical applications (usually milliseconds are required). You should optimize the code or admit this limitation. The heavy reliance on trig functions and matrix reshaping is likely the bottleneck. Please clarify or revise.

**ANS:** Inspired by your valuable suggestions, we have optimized the encryption algorithm accordingly and retested it under the same experimental conditions. Currently, the encryption time for a 1024×1024 image has been reduced to approximately 4.2 seconds, representing a significant improvement over the original version. We fully acknowledge that encryption efficiency in practical applications typically needs to reach the millisecond level, and there remains room for further optimization in current performance. The speed of image encryption is influenced by multiple factors, including algorithm implementation efficiency, hardware conditions, and image size characteristics. In our future work, we will continue to focus on optimizing both the algorithm and the code to further enhance the real-time performance and practicality of the encryption process.

10. The text mentions changing “one bit” for NPCR/UACI tests. Did you test changing bits in different positions (MSB vs. LSB)? Chaotic systems can sometimes be less sensitive to LSB changes in floating-point domains. A more comprehensive sensitivity test across different bit planes would be convincing.

**ANS:** In the NPCR/UACI tests against differential attacks, we have systematically evaluated the sensitivity of the algorithm to changes in different bit positions. Specifically, modifications were tested for the Least Significant Bit (LSB, i.e., bit 0), the middle bit (bit 3), and the Most Significant Bit (MSB, i.e., bit 7). The experimental results show that the encryption system exhibits good sensitivity across all tested bit planes, with no observed insensitivity to LSB changes in floating-point arithmetic environments. This confirms that the proposed algorithm possesses comprehensive and reliable differential sensitivity.

11. The direction of diffusion (clockwise/counter-clockwise) is determined by the random number X. Since X is derived from the key/hash, it is deterministic. Does this really add security complexity, or is it just a linear variation? Please discuss the cryptanalytic benefit of this bi-directional feature.

**ANS:** Indeed, in our algorithm, the diffusion direction (clockwise/counterclockwise) is determined by the random variable X. X is generated through iterative computation from the input initial value and the hash value of the plain image, producing a chaotic sequence. Clockwise and counterclockwise represent two distinct diffusion directions. By randomly selecting between these directions based on the chaotic sequence, the complexity of the algorithm is effectively increased, thereby enhancing security.

12. In Table 3 and Table 4, you compare your results with references from 2024 and 2025. Ensure that the test images used in those references are identical (same dimensions and source) to yours. If dimensions differ, metrics like NPCR and local entropy cannot be directly compared.

**ANS:** We have rechecked and confirmed that the benchmark test images used for comparative analysis in the manuscript are consistent in both size and data source, thereby ensuring the comparability of the data.

13. A well-crafted abstract should concisely present the research background, identify the research gap, highlight the study’s novel contributions, summarize key findings, particularly critical experimental results, and emphasize the work’s broader significance and value. Please refer to State-dependent variable fractional-order hyperchaotic dynamics in a coupled quadratic map: a novel system for high-performance image protection and Dynamics analysis, synchronization and FPGA implementation of multiscroll Hopfield neural networks with non-polynomial memristor for improvements.

**ANS:** Inspired by your suggestions, we have carefully revised the abstract as follows:

As color images have become a cornerstone of information exchange in the digital age, ensuring their security is of paramount importance. Departing from traditional scrambling–diffusion structures, this paper foregrounds a novel color image encryption algorithm by proposing the integration of an ∞-shaped transformation with a closed-loop control mechanism. The specific implementation process is as follows: first, the three channel matrices are merged, and the elements in each row are linked into a closed loop for initial diffusion. The diffused image is then restructured into a three-row matrix and scrambled using a unique ∞-shaped transformation. Finally, column-wise closed-loop diffusion is applied to generate the cipher image. This algorithm not only achieves effective inter-channel pixel confusion through the ∞-shaped transformation, but also performs diffusion and confusion under the closed-loop control model. Experimental results demonstrate the algorithm’s excellent overall performance: the key space is as large as 2⁴¹³, information entropy approaches the ideal value of 8 as image size increases, and the algorithm exhibits high sensitivity, with NPCR and UACI exceeding 99.6% and 33.4%, respectively. Quantitative evaluation confirms that the proposed scheme offers strong robustness against differential, statistical, and brute-force attacks.

14. The current keyword list does not fully capture the core content and distinctive contributions of the manuscript. The author is encouraged to revise it to better align with the paper’s key themes and technical focus. Please refer to Two-dimensional coupling-enhanced cubic hyperchaotic map with exponential parameters: construction, analysis, and application in hierarchical significance-aware multi-image encryption and Exploiting dynamic vector-level operations and a 2D-enhanced logistic modular map for efficient chaotic image encryption for revisions. For example, the author should consider adding keywords such as “Dynamical analysis” and “security analysis”.

**ANS:** Inspired by your suggestions, we have updated the keywords to better reflect the core contributions and technical focus of this study. The revised list is provided below: Color image encryption; Hyperchaotic system; ∞-shaped transformation; Closed-loop diffusion; Security analysis.

15. Given that your work focuses on chaotic encryption, the Introduction should provide a systematic and categorized overview of recent chaotic encryption methods based on new techniques (neural networks, quantum computing, etc.), such as Dynamics analysis, synchronization and FPGA implementation of multiscroll Hopfield neural networks with non-polynomial memristor, State-dependent variable fractional-order hyperchaotic dynamics in a coupled quadratic map: a novel system for high-performance image protection, Two-dimensional coupling-enhanced cubic hyperchaotic map with exponential parameters: construction, analysis, and application in hierarchical significance-aware multi-image encryption, and Exploiting dynamic vector-level operations and a 2D-enhanced logistic modular map for efficient chaotic image encryption. Please refer to the first paragraph of Exploiting robust quadratic polynomial hyperchaotic map and pixel fusion strategy for efficient image encryption for revisions.

**ANS:** Inspired by your suggestions, I have carefully studied the latest advancements in the field and revised the opening paragraph of the introduction in the paper.

16. Cryptanalysis targeting image encryption holds significant reference value for designing encryption schemes. The authors should incorporate recent cryptanalysis studies in the introduction, such as Cryptanalysis of an image encryption cryptosystem based on binary bit planes extraction and multiple chaotic maps, Security analysis of a color image encryption algorithm using a fractional-order chaos, and Cryptanalysis and improvement of the image encryption scheme based on Feistel network and dynamic DNA encoding. Besides, the authors should evaluate whether the proposed work has sufficient practicality and security based on these cryptanalysis works.

**ANS:** As suggested, we have revised the introduction to include recent advances in image encryption cryptanalysis. This provides a more robust context for our work and better demonstrates the practical motivation and security considerations behind the proposed algorithm.

17. There are some issues with the presentation and explanation of mathematical symbols and equations. Each equation should be followed by a comma or period, as appropriate. Please refer to State-dependent variable fractional-order hyperchaotic dynamics in a coupled quadratic map: a novel system for high-performance image protection and Dynamics analysis, synchronization and FPGA implementation of multiscroll Hopfield neural networks with non-polynomial memristor for improvements.

**ANS:** We have carefully reviewed the recommended literature. Following these examples, we have thoroughly examined and revised the entire manuscript to ensure that a comma or period is correctly placed after each formula, that all mathematical symbols are clearly defined upon their first use, and that explanatory texts remain consistent and precise. We believe these revisions have significantly enhanced the clarity and academic rigor of the paper.

18. The conclusion feels underdeveloped and lacks impact. I suggest the authors restructure it, drawing inspiration from Two-dimensional coupling-enhanced cubic hyperchaotic map with exponential parameters: construction, analysis, and application in hierarchical significance-aware multi-image encryption and Exploiting dynamic vector-level operations and a 2D-enhanced logistic modular map for efficient chaotic image encryption, to clearly highlight the study’s core findings, main contributions, existing challenges, proposed solutions, key limitations, and future directions in a more compelling and cohesive way.

**ANS:** Inspired by your suggestions, we have reviewed relevant literature and revised the summary section of the paper.

19. Please revise and enhance the reference list. Specifically, remove low-quality sources (e.g., papers published in non-SCI journals such as Multimedia Tools and Applications or non-prestigious conferences) and outdated references. Instead, incorporate, introduce, analyze, discuss, and compare the most recent relevant works to strengthen the research background, particularly the discussion of chaotic image encryption and its cryptanalysis studies.

**ANS:** Thank you for your suggestion. In accordance with your feedback, we have thoroughly reviewed and updated the references.

**Response to Reviewer 2’s Comments**

Firstly, we would like to express our sincere thanks for your constructive comments and positive response. The revised version of the manuscript has been significantly modified based on your comments. The revised parts are marked in red font. We hope you are satisfied with our revisions.

1. What is the research gap of this paper?

**ANS:** This study focuses on the following key innovations: (1) While most existing algorithms design the diffusion and scrambling stages separately, this paper introduces a closed-loop control mechanism that enables simultaneous scrambling and diffusion, thereby significantly enhancing the algorithm’s complexity and security. (2) Conventional transformations such as Zigzag are typically limited to intra-channel scrambling operations. In contrast, the novel “∞-shape transformation” proposed in this paper possesses a unique structure that not only allows synchronous cross-channel data permutation but also enables dynamic scrambling, further increasing the complexity of the scrambling process.

In summary, the contribution of this study lies in the development of a novel, efficient, and highly secure color image encryption framework. By integrating the ∞-shape transformation with the closed-loop control mechanism, this framework achieves efficient confusion and diffusion in a single processing flow while maintaining excellent encryption performance even for large-scale images. Experimental results demonstrate that the proposed algorithm exhibits outstanding performance in terms of key space, information entropy, and resistance to differential attacks, offering a new and viable solution for color image encryption.

2. What are the theoretical advantages of ∞-shaped over U-shaped, Z-shaped, or Hilbert-shaped? And is it just a different traversal path, or does it have new cryptographic properties?

**ANS:** The ∞-shape transformation represents a novel structure distinct from U-shaped, Z-shaped, or Hilbert-shaped transformations. First, it employs an inherent three-row matrix transformation to achieve direct cross-channel confusion among the three color components. This not only disrupts the correlation between adjacent pixels in the original image but also effectively reduces inter-channel pixel correlations simultaneously. Second, the transformation can adopt five different morphological patterns based on the number of columns, which further enhances the complexity of the algorithm through dynamic scrambling effects.

To verify the effectiveness of the ∞-shaped transform compared to classical scrambling methods such as Zigzag, simulation experiments were conducted using a 256×256 image “house”, with the results presented in Table 3. It presents a comparative analysis of correlation coefficients for both adjacent pixels and inter-channel pixels under different scrambling schemes. It can be observed that the ∞-shaped transform outperforms the Hilbert transform, Zigzag transform, and Peano curve transform in disrupting both spatial correlations among neighboring pixels and statistical dependencies across color channels. These results demonstrate the superior decorrelation capability and scrambling randomness of the ∞-shaped transform. In contrast, the other scrambling methods exhibit limited effectiveness in suppressing inter-channel correlation, with some cases showing negligible impact. Overall, the ∞-shaped transform achieves more comprehensive performance in image scrambling

tasks.

**Table 3.  Comparison of correlation coefficients of different scrambling schemes.**

| **Scrambling Path** | | | **Original**  **image** | **Hilbert transform** | **Zigzag transform** | **Peano curve transform** | **∞-shaped transform** |
| --- | --- | --- | --- | --- | --- | --- | --- |
| **Adjacent Pixel Correlation** | R | H | 0.9671 | 0.6984 | 0.1980 | 0.7679 | 0.6638 |
|  |  | V | 0.9353 | 0.6975 | 0.1977 | 0.6504 | 0.6696 |
|  |  | D | 0.9126 | 0.5613 | 0.1661 | 0.5504 | 0.5659 |
|  | G | H | 0.9805 | 0.7614 | 0.1669 | 0.7899 | 0.7685 |
|  |  | V | 0.9474 | 0.7598 | 0.1674 | 0.7247 | 0.7643 |
|  |  | D | 0.9320 | 0.6367 | 0.1478 | 0.6240 | 0.5254 |
|  | B | H | 0.9820 | 0.8190 | 0.2039 | 0.8404 | 0.7258 |
|  |  | V | 0.9749 | 0.8180 | 0.2055 | 0.7881 | 0.7318 |
|  |  | D | 0.9625 | 0.7101 | 0.2061 | 0.6903 | 0.7404 |
| **Inter-channel Pixel Correlation** | R-G | | 0.6378 | 0.6378 | 0.6378 | 0.6378 | 0.5420 |
|  | R-B | | 0.4823 | 0.4823 | 0.4823 | 0.4823 | 0.6300 |
|  | G-B | | 0.9418 | 0.9418 | 0.9418 | 0.9418 | 0.6499 |

3. Discussion image encryption is very low, i suggest author discuss some new work like“https://doi.org/10.1038/s41598-024-80969-z”, “https://doi.org/10.1109/ACCESS.2024.3351693” and “https://doi.org/10.1109/ACCESS.2020.3011724”

**ANS:** Thank you for your suggestion. In accordance with your feedback, we have revisited the relevant literature and updated the manuscript accordingly.

4. The manuscript over-emphasizes chaotic dynamics analysis without clearly linking them to practical cryptographic strength.

**ANS:** Experimental results demonstrate that the proposed chaotic system performs well and has been successfully applied to image encryption. The analytical metrics of the chaotic system—such as the Lyapunov exponent, bifurcation diagram, and phase portrait—are closely related to cryptographic strength evaluation indicators, as detailed below:

(1) Key sensitivity and diffusion property – The high positive Lyapunov exponent of the chaotic mapping ensures extreme sensitivity to initial conditions. This is reflected in the encryption scheme by high NPCR (>99.6%) and UACI (>33.4%) values, even with minimal changes to the key.

(2) Randomness and statistical security – The ergodicity and broadband entropy of the chaotic mapping contribute to cipher images with near‑ideal information entropy (approximately 8) and a uniform histogram, effectively resisting statistical attacks.

(3) Large key space – The wide chaotic parameter range shown in the bifurcation diagram corresponds to a key space of 2^413^, which provides strong resistance against brute‑force attacks.

In summary, chaos analysis is not presented in isolation; rather, it serves as a theoretical foundation that directly links to and supports the practical cryptographic strength of the encryption algorithm.

5. Compare cipher-image entropy with and without chaos.

**ANS:** Kerckhoffs’s Principle serves as a fundamental criterion in the design of cryptographic systems, which asserts that the security of a system should rely entirely on the secrecy of the key and not on the obscurity of the algorithm. The proposed algorithm in this study employs the initial values of chaos as the key, iteratively generating chaotic sequences to serve as pseudo‑random sequences in the encryption process. As shown in Table 5, the variation in information entropy before and after encryption confirms the algorithm's effectiveness. The chaotic sequence serves as the essential key; without it, the algorithm would violate Kerckhoffs’s Principle and its theoretical security would collapse.

6. I suggest author add Cipher-image entropy vs u, r parameters.

**ANS:** In the proposed algorithm, the parameters u and r of the chaotic map are both derived from the hash value of the plain image via Algorithm 1. That is, for different images, distinct values of u and r—along with the corresponding chaotic initial values—are computed from the input key and then iterated to generate the chaotic sequence used for encryption. The resulting information entropy values for the different encrypted images are provided in Table 5.

7. Attacker capability not explained: known-plaintext? chosen-ciphertext?

**ANS:** We have now clarified the attacker capability in the revised manuscript as follows:

(1) Attack Model: Our security analysis primarily focuses on the chosen-plaintext attack (CPA) model, where an attacker can select arbitrary plain images to encrypt. We assume the attacker has access to the encryption machinery but does not know the secret input key.
(2) CPA Analysis: As presented in Section 5.3, we specifically used all-black and all-white images as chosen plaintexts to test the algorithm. The results show that their corresponding ciphertexts are uniformly distributed noise images with no statistical similarity to the inputs. This confirms that the scheme does not leak any information about the secret key under CPA.
(3) KPA Implication: For known-plaintext attacks (KPA), since the encryption key is derived from both the plaintext hash and a secret input key, possessing a plaintext-ciphertext pair does not reveal the secret input key due to the one-way property of the hash function.

8. What the attacker knows? What is hidden? and Is SHA-384 enough to prevent known-plaintext attacks?

**ANS:** 8.1 What the attacker knows? What is hidden?

According to Kerckhoffs’s principle, we assume that the attacker may know the encryption algorithm and may have obtained the encrypted image, but the specific key remains unknown.

8.2 Is SHA-384 enough to prevent known-plaintext attacks?

Yes. In our algorithm, SHA-384 is employed not as the core encryption cipher, but as a key derivation function. It computes the hash value of the plain image to serve as an initial seed, which, combined with the user input key, generates the key for the encryption chaotic system. Consequently, even a slight change in either the plain image or the user’s initial input key results in a completely different chaotic sequence. The primary defense against known-plaintext attacks in this scheme relies on the high sensitivity of the chaotic system and a structured diffusion-confusion layer (implementing ∞-shaped transformation and closed-loop diffusion). SHA-384 is used solely to ensure sufficient initial entropy for the chaotic system. Even if an attacker obtains some plaintext-ciphertext pairs, the one-way nature of SHA-384 and the iterative mechanism of the chaotic system make it computationally infeasible to deduce the chaotic seed or the original user key.

9. Robustness against noise and cropping is not a standard security requirement for encryption algorithms and may contradict cryptographic diffusion principles.

**ANS:** Robustness against noise and cropping is not a standard security requirement for encryption algorithms and indeed appears contradictory to the principle of diffusion in cryptography. In practical algorithm design, we strive to balance the need for strong diffusion with a degree of tolerance to common image processing disturbances, aiming to achieve an effective trade-off between the two.

10. Algorithmic complexity analysis is missing

**ANS:** In response to your suggestions, we have added Section 5.10 “Algorithmic Complexity Analysis” to the paper, providing a detailed examination primarily from the perspective of time complexity. The analysis reveals that the overall time complexity of the proposed encryption scheme is O( M× N), i.e., linear time complexity.

11. Check typo and grammatical error

**ANS:** We have carefully reviewed and revised the grammar and spelling throughout the manuscript. The text has also been polished with the assistance of colleagues from the College of Foreign Languages and further reviewed collectively by our research team.

**Response to Reviewer 3’s Comments**

Firstly, we would like to express our sincere thanks for your constructive comments and positive response. The revised version of the manuscript has been significantly modified based on your comments. The revised parts are marked in red font. We hope you are satisfied with our revisions.

1. Proposed 2D-CSHS chaotic map is mathematically defined and experimentally analyzed, but its novelty over existing coupled sine–cosine or logistic-based maps is not sufficiently justified. A direct analytical or experimental comparison with closely related 2D hyperchaotic maps is required to clarify what new dynamical property or security advantage this map uniquely provides.

**ANS：**Comparative experiments were conducted in this study, in which the

parameters of each map were normalized. As shown in Fig 5, 2D-CSHS exhibits a continuous chaotic parameter range, and its LLE is significantly higher than those of the other maps under comparison. In other words, 2D-CSHS outperforms the other

maps in terms of the Lyapunov exponent.

**Fig 5. LLE comparison for 2D- CSHS and four other proposed 2D maps**

2. Bifurcation, Lyapunov exponent, and sample entropy analyses confirm chaotic behavior, but these results are largely descriptive. The paper should explain how the chosen parameter ranges specifically impact encryption strength, rather than only stating that they exhibit chaos. A short sensitivity discussion linking chaotic metrics to cryptographic performance would improve rigor. Next,the ∞-shaped transformation is visually intuitive, but its security role is not formally analyzed. The paper should discuss whether this transformation provides stronger mixing than existing scan-based permutations such as Hilbert, zigzag, or U-shaped scans, especially in terms of inter-channel decorrelation. A quantitative comparison would strengthen this section.

**ANS:** 2.1 Experimental results demonstrate that the proposed chaotic system performs well and has been successfully applied to image encryption. Importantly, the selected parameter ranges directly determine cryptographic strength, as evidenced by the

following links between chaos metrics and security indicators:

(1) Key sensitivity – The high positive Lyapunov exponent (ensured by the chosen parameter range) guarantees extreme sensitivity to initial conditions, which translates

into NPCR > 99.6% and UACI > 33.4% under minor key changes.

(2) Statistical security – The bifurcation diagram confirms that the operating parameters lie in a fully chaotic region, ensuring ergodicity. This yields cipher images with entropy approaching 8 and uniform histograms, effectively resisting statistical

attacks.

(3) Key space – The wide chaotic parameter range shown in the bifurcation diagram expands the key space to 2⁴¹³, providing robust resistance against brute-force

attacks.

In summary, the chaos analysis is not merely descriptive; it establishes how the

chosen parameter ranges underpin the algorithm's cryptographic strength.

2.2 To verify the effectiveness of the ∞-shaped transform compared to classical scrambling methods such as Zigzag, simulation experiments were conducted using a 256×256 image “house”, with the results presented in Table 3. It presents a comparative analysis of correlation coefficients for both adjacent pixels and inter-channel pixels under different scrambling schemes. It can be observed that the ∞-shaped transform outperforms the Hilbert transform, Zigzag transform, and Peano curve transform in disrupting both spatial correlations among neighboring pixels and statistical dependencies across color channels. Specifically, this scheme substantially reduces the horizontal, vertical, and diagonal correlations of the R, G, and B channels, while also effectively weakening the inter-channel correlations (e.g., R–G, R–B, G–B). These results demonstrate the superior decorrelation capability and scrambling randomness of the ∞-shaped transform. In contrast, the other scrambling methods exhibit limited effectiveness in suppressing inter-channel correlation, with some cases showing negligible impact. Overall, the ∞-shaped transform achieves more

comprehensive performance in image scrambling tasks.

**Table 3.  Comparison of correlation coefficients of different scrambling schemes.**

| **Scrambling Path** | | | **Original**  **image** | **Hilbert transform** | **Zigzag transform** | **Peano curve transform** | **∞-shaped transform** |
| --- | --- | --- | --- | --- | --- | --- | --- |
| **Adjacent Pixel Correlation** | R | H | 0.9671 | 0.6984 | 0.1980 | 0.7679 | 0.6638 |
|  |  | V | 0.9353 | 0.6975 | 0.1977 | 0.6504 | 0.6696 |
|  |  | D | 0.9126 | 0.5613 | 0.1661 | 0.5504 | 0.5659 |
|  | G | H | 0.9805 | 0.7614 | 0.1669 | 0.7899 | 0.7685 |
|  |  | V | 0.9474 | 0.7598 | 0.1674 | 0.7247 | 0.7643 |
|  |  | D | 0.9320 | 0.6367 | 0.1478 | 0.6240 | 0.5254 |
|  | B | H | 0.9820 | 0.8190 | 0.2039 | 0.8404 | 0.7258 |
|  |  | V | 0.9749 | 0.8180 | 0.2055 | 0.7881 | 0.7318 |
|  |  | D | 0.9625 | 0.7101 | 0.2061 | 0.6903 | 0.7404 |
| **Inter-channel Pixel Correlation** | R-G | | 0.6378 | 0.6378 | 0.6378 | 0.6378 | 0.5420 |
|  | R-B | | 0.4823 | 0.4823 | 0.4823 | 0.4823 | 0.6300 |
|  | G-B | | 0.9418 | 0.9418 | 0.9418 | 0.9418 | 0.6499 |

3. The closed-loop control model combines scrambling and diffusion, which is a good design choice. However, the diffusion equations are complex and not easy to verify. A simplified explanation of how error propagation spreads across the loop would help readers understand why this design improves resistance to differential attacks. Also, the key generation process relies heavily on SHA-384 and chaotic iterations, leading to a very large key space. However, the practical independence between hash-derived keys and chaotic parameters is not clearly proven. The authors should clarify whether any correlation exists between these key components under finite precision arithmetic.

**ANS:** 3.1 As shown in Equations (7) and (8), during the diffusion stage, the value of each pixel is updated based on the previously encrypted output from one or even two preceding steps. This cyclic dependency within the closed‑loop structure establishes an error propagation mechanism: even a minute change in the input image (e.g., a difference in a single pixel) is continuously transmitted, accumulated, and diffused throughout the entire image through successive iterations in the closed loop. Consequently, this design significantly enhances the algorithm’s resistance to differential attacks.

3.2 The hash-derived keys and the chaotic parameters are mutually independent and uncorrelated. The specific calculation process can be found in lines 1–14 of Algorithm 1.

4. Finite State Machine based image encryption schemes model the cipher as a finite number of states with deterministic or key-controlled state transitions. While this structure is simple and efficient, the state space is inherently limited. Once the transition function and output mapping are inferred, the system behavior becomes predictable. So, Authos may compare some papers like Enhancing image security via block cyclic construction and DNA based LFSR; Chaos-based medical image encryption scheme using special nonlinear filtering function based LFSR; PwLMμ-TPE: A Visual-Usability and Privacy-Enhanced Thumbnail Preserving Encryption Scheme of Cloud Computing for Consumers. Authors are informed to include a clear security comparison with FSM-based encryption schemes, highlighting how the proposed chaotic and closed-loop diffusion approach avoids finite state predictability and offers stronger resistance to state reconstruction and differential attacks.

**ANS:** We have carefully studied the relevant literature on finite-state machine (FSM)-based encryption schemes and gained a clear understanding of their structural characteristics and potential limitations. Accordingly, we have updated both the introduction and the algorithm analysis sections to explicitly explain how the proposed chaotic-driven, closed-loop diffusion mechanism avoids the predictability inherent in finite-state systems, thereby theoretically enhancing the algorithm’s resistance to state reconstruction attacks and differential attacks.

5. Insecurity analysis, most metrics are close to ideal values, but comparisons are limited to a small set of reference schemes. Including at least one lightweight or low-complexity encryption scheme as a baseline would make the performance claims more balanced and realistic.The robustness analysis under noise and cropping shows visually acceptable decrypted images, but the security implication of partial information recovery is not discussed. From a cryptographic perspective, successful recovery after severe cropping may also indicate diffusion weakness, which should be critically analyzed.

**ANS:** 5.1 Following your suggestion, we have included several lightweight and low-complexity encryption schemes as baselines in the comparative analysis, making

the performance evaluation more comprehensive and objective.

5.2 Your observation is very pertinent. There is an inherent tension between robustness against noise/cropping and the diffusion principle in cryptography. The fact that meaningful information can still be recovered after severe cropping may indicate the presence of residual structural redundancy in the image, which deviates from the ideal diffusion requirement that “a change in one bit affects the entire output.” In our practical algorithm design, we attempt to strike a balance between the security provided by strong diffusion and a reasonable level of tolerance to common image manipulations (e.g., transmission noise, cropping). Future work will focus on exploring how to enhance the completeness of diffusion while maintaining necessary robustness, thereby better meeting rigorous security requirements.
